# Exercise-Induced Changes in Iron Status and Hepcidin Response in Female Runners

**DOI:** 10.1371/journal.pone.0058090

**Published:** 2013-03-05

**Authors:** Irena Auersperger, Branko Škof, Bojan Leskošek, Bojan Knap, Aleš Jerin, Mitja Lainscak

**Affiliations:** 1 Faculty of Sport, University of Ljubljana, Ljubljana, Slovenia; 2 Department of Nephrology, University Medical Centre Ljubljana, Ljubljana, Slovenia; 3 Institute of Clinical Chemistry and Biochemistry, University Medical Centre Ljubljana, Ljubljana, Slovenia; 4 Division of Cardiology, University Clinic Golnik, Golnik, Slovenia; 5 Applied Cachexia Research, Department of Cardiology, Charité, Campus Virchow-Klinikum, Berlin, Germany; Indiana University, United States of America

## Abstract

**Background and Aims:**

Exercise-induced iron deficiency is a common finding in endurance athletes. It has been suggested recently that hepcidin may be an important mediator in this process.

**Objective:**

To determine hepcidin levels and markers of iron status during long-term exercise training in female runners with depleted and normal iron stores.

**Methods:**

Fourteen runners were divided into two groups according to iron status. Blood samples were taken during a period of eight weeks at baseline, after training and after ten days’ recovery phase.

**Results:**

Of 14 runners, 7 were iron deficient at baseline and 10 after training. Hepcidin was lower at recovery compared with baseline (p<0.05). The mean cell haemoglobin content, haemoglobin content per reticulocyte and total iron binding capacity all decreased, whereas soluble transferrin receptor and hypochromic red cells increased after training and recovery (p<0.05 for all).

**Conclusion:**

The prevalence of depleted iron stores was 71% at the end of the training phase. Hepcidin and iron stores decreased during long-term running training and did not recover after ten days, regardless of baseline iron status.

## Introduction

Athletes, particularly females and adolescents, are at increased risk of depleting their iron stores to the stage of functional or absolute iron deficiency (ID). If unrecognized or untreated, sideropenic anaemia may eventually develop [1].The prevalence of ID in adolescent and adult females competing in a variety of sports was reported to range from 25% to 36% [2], [3]. During the season this can change significantly and ranges from 14% to 70% [4]. Inadequate body iron stores may decrease physical performance to present as unusual fatigue, exercise intolerance and even as distinct cognitive impairment [5], [6].

Several indicators can be used to evaluate iron store, serum ferritin concentration being the most common [2]. A 20 µg/L threshold for ID was widely accepted in previous studies in athletes [2], [3], [7]. Recently, the serum concentration of soluble transferrin receptor (sTfR) and the ratio of sTfR to serum ferritin (sTfR-F index) have been suggested as more sensitive indicators of functional ID or tissue iron depletion over serum ferritin alone [2], [6].

Exercise-induced mechanisms of iron loss are haemolysis, haematuria, sweating and gastrointestinal bleeding [8]–[10]. A major advance in recent years was the identification of hepcidin, an acute-phase reactant, as a key regulator of whole-body iron homeostasis. Because number of physiological triggers stimulate hepcidin expression (e.g. inflammation, hypoxia, elevated iron levels), it is likely that hepcidin acts beyond the acute phase [11].

Only a few studies have investigated the acute post-exercise kinetics of hepcidin and associations with iron metabolism in athletes [12]–[14]. Most report about elevated hepcidin levels 24 h after exercise, preceded by acute increase of serum iron and inflammation parameters [12], [13], [15]. An individual response, however, has to be considered, as athletes with ID demonstrated an attenuated response of hepcidin [15], [16]. The effects of regular repetitive exercise have been less well studied. Karl et al [17] showed that nine weeks of basic combat training (BCT) affected iron status in female soldiers, but not hepcidin. Serum hepcidin concentrations were associated with iron status, lower in soldiers with iron deficiency anaemia (IDA). Positive relation with serum ferritin concentrations and inflammation was found.

In view of the limited data, particularly in females, we conceived a study to investigate hepcidin and iron status markers during long-term exercise training in female runners with depleted and normal iron stores. We also evaluated the effects of short recovery phase on iron stores.

## Materials and Methods

### Participants

This was a prospective observational study which screened moderately physically active females (3 or 4 sessions/week) in reproductive age and without histoy of gynecological surgery (5 participants reported up to 2 pregnancies, all were uneventful). Participants were eligible for inclusion in the study if they met the following criteria: regular menstrual cycles (defined as 9–12 menstrual cycles per year with cycles occurring at regular intervals); regular dietary intake of animal products (defined as intake of at least one serving of meat, fish or poultry on most days); no use of iron supplements; no regular use of medication, except oral contraceptives. All women with anaemia (haemoglobin [Hb] <120 g/L) were excluded from the study. Finally, 14 participants were enrolled and were classified into two groups according to their iron stores: ferritin levels >20 µg/L (normal iron stores: group N, 7 participants); ferritin levels ≤20 µg/L (depleted iron stores: group D, 7 participants) (Table 1). According to detailed disease history, physical examination prior to study begin, and baseline laboratory analysis, no acute or chronic inflammatory condition was present in any of the participants. The study protocol was approved by the National Ethics Committee of Slovenia. After being informed of the purpose, potential benefits and possible risks of the study protocol, all participants gave written informed consent prior to any study-related procedure.

**Table 1:** Mean (± SD) baseline and recovery characteristics of 14 female runners: group N = baseline serum ferritin (SF)>20 µg/L, group D = baseline SF≤20 µg/L.

Group	Time	Age	Weight	Height	Fat free mass	Fat	VO2 max	Cooper test
		(year)	(kg)	(cm)	(kg)	(%)	(mL/kg* min)	(s)/2400 m
**Group N**	**Baseline**	31.4	61.6	169.7	50.6	19.9	45.6	681.0
**n = 7**		(5.9)	(7.7)	(5.2)	(4.1)	(3.3)	(2.8)	(75.5)
	**Recovery**		60,9		50.0	19.3	47.8^a^	638.9^a^
			(8.4)		(4.4)	(3.9)	(3.5)	(72.0)
**Group D**	**Baseline**	34.9	58.4	164.8	48.1	19.3	47.4	710.3
**n = 7**		(4.7)	(9.1)	(5.9)	(5.5)	(3.4)	(4.7)	(89.6)
	**Recovery**		58.0		47.6	19.1	48.8	674.6^a^
			(7.7)		(4.5)	(3.7)	(5.9)	(91.0)

a significantly different from baseline value (p<0.05).

### Experimental Protocol

The study was completed during an intensified training phase, focusing on competitive 10 km or 21 km runs in the International Ljubljana Marathon, October 2008. Before beginning the training process, all runners completed a two-week run-in period of low-intensity physical training to ensure familiarity with experimental procedures and to have reached a non-fatigued state. At the beginning of the experimental training period, which lasted for eight weeks and at the end of experiment (one week after the race), all runners completed incremental tests to exhaustion, ran a 2400-m time trial (Cooper test) on an outdoor 400 m Tartan track and had their anthropometric status measured.

The physical training program consisted of two three-week progressive overload periods each followed by a one week taper. After the second week taper the runners participated in the race. Blood samples were taken at three time-points: before the training program (Baseline), after completion of training (Training), and after ten days’ recovery phase (Recovery).

The participants had three or four training session per week in training load phases consisting of one or two interval trainings (one at 88–95% maximum heart rate [MHR], the second up to 100% MHR) and two easy runs (at 70–87% MHR) of 6–8 km and 12–18 km. Training volume was matched with the anticipated race distance. We set each runner’s training protocol to match the same training stimulus based on runner MHR determined on baseline incremental testing to exhaustion. Tapering-period interval training was replaced with an easy run of 6–8 km, three times per week. All training sessions were supervised by at least one qualified athlete coach and one member of the research group.

### Measurements

Anthropometric measurements were performed at baseline and at recovery. Body weight (kg) and height (cm) were measured to the nearest 0.1 kg and 0.5 cm, respectively. Body fat percentage (%) and lean mass (kg) were assessed using the skinfold technique and calculated using Matiegka’s method. Skinfold thicknesses at biceps, triceps and subscapular were measured with GPM skinfold callipers (Siber Hegner & Co. Ltd., Zurich, Switzerland) with a precision of 0.2 mm.

All participants had previous experience of treadmill running and completed incremental treadmill tests to exhaustion at baseline and recovery phase. After a 6-min warm-up, an incremental protocol on a calibrated treadmill (Technogym, UK) with a 2% incline was applied. The starting speed was 3 km/h with speed increments of 2 km/h every 2 min. The runners walked the first stage and then ran until volitional exhaustion. The last half or full stage that the participant could sustain (for either 1 min or 2 min) was defined as that individual’s maximal speed. Maximum oxygen consumption (VO_2_ max) was assessed using a Cosmed K4b2 (Rome, Italy) spirometric system. Heart rate (HR) was recorded continuously during the test using telemetric heart monitors (Polar Electro, Oulu, Finland) and data stored on a computer.

To record and control training, each participant was equipped with a HR monitor (Group I, Polar RS800sd; Group C, Polar RS400sd, Finland) during the process, not including warm-up and cool-down intervals but noting the recovery interval on interval training days. Weekly exercise scores were calculated from the training loads using a method based on the HR zone training point system (∑ HR) [18].The length of time (in min) within various HR-based zones was computed from the HR monitor, multiplied by the value of the zone, and summated to derive ∑ HR zone training points. The HR zones were based on <70% (zone 1 =  value 1), 71–79% (zone 2 = 2), 80–87% (zone 3 = 3), 88–93% (zone 4 = 4) and >94% (zone 5 = 5) of the previously established MHR for that mode of exercise in that particular individual [19].

Blood samples were collected after an overnight fast, between 7 am and 8 am to avoid variations in circadian rhythms, and 24 h after exercise. All participants were asked to refrain from drinking coffee, tea, chocolate or cola drinks, and to avoid alcohol for that 24 h period. All blood samples were taken with the participant in a seated position. EDTA blood samples were sent for immediate analysis of red blood cells concentration and distribution width, Hb, reticulocytes, leukocytes and platelets concentration on an Advia 2010 analyzer (Siemens Healthcare, Erlangen, Germany). For analyses of serum iron (Fe), total iron binding capacity (TIBC), ferritin, transferrin, soluble transferrin receptors (sTfR), C-reactive protein (CRP), interleukin-6 (IL-6) and hepcidin, blood samples were collected without additive; after centrifugation sera were stored at −20°C. Iron and TIBC were measured spectrophotometrically in an Advia 1800 analyzer (Siemens Healthcare, Erlangen, Germany) and ferritin was measured by immunoturbidimetric assay in an Olympus AU400 analyzer (Beckman Coulter, CA, USA). Transferrin and TfR were measured using immunonephelometry on the BN System II (Siemens Healthcare, Erlangen, Germany). CRP was measured using a chemiluminescent immunometric high-sensitivity assay with a detection limit of 0.3 mg/L (Immulite analyzer; Siemens Healthcare, Erlangen, Germany) and IL-6 was measured by electrochemiluminescence assay with a detection limit of 2 ng/L (Cobas e411 analyzer, Roche Diagnostics, Mannheim, Germany). Hepcidin was measured in an ELISA with an analytical sensitivity of 4 µg/L (IBL, Hamburg, Germany, reference values 59–158 µg/L), as described previously [20].

In this study, groups were primary classified by serum ferritin 20****µg/L threshold into group N and group D as defined previously [7], [21]. We also determined the prevalence of ID in accordance with other studies in athletes and female reference levels [2], [3], [22]. For this purpose, participants were classified into Category N = normal iron stores defined as serum ferritin >30 µg/L and Category D = depleted iron stores defined as serum ferritin <30 µg/L, both with normal Hb>120 g/L and transferrin saturation (TSAT; 20–40%); Category ID =  determined by serum ferritin <12 µg/L and TSAT<16%; Category IDA = who met the criteria for category ID and presented Hb<120 g/L [21]. The sTfR-F index (transferrin receptor: log serum ferritin ≥1.5) was used as an alternative determination of ID without anemia [23], [24].

### Statistics

Results are expressed as mean ± standard deviation (SD). Differences between groups in baseline values of anthropometric measurements, VO_2_max and results of Cooper tests (2400-m time trial) were analyzed with Mann–Whitney tests. The effects of group, times, the interaction of these on exercise intensity, and laboratory markers were analyzed with repeated measures ANOVA. Analysis of contrasts was used to determine where specific pre-planned differences existed. Assumption of normality was inspected with histograms and Q-Q plots, and logarithmic transformation was applied to adjust for non-normality where appropriate. A p value <0.05 was considered statistically significant. SPSS (IMB) PASW Statistics 18.0 was used for the analysis.

## Results

### Physical Responses to Training

The study included 14 participants and all completed the study protocol as scheduled. As shown in Table 1, there were no significant baseline differences between the two groups in age, height, body weight, % of fat mass, lean mass, Cooper test or VO_2_max. We did not observe any significant differences in body composition between baseline and recovery. At completion of the programme improvement of physical performance was observed in both groups: the average increase in VO_2_max from baseline to recovery was +4.8% in group N and +3% in group D, but was significant only in group N. The time during the Cooper test was significantly shorter (group N, −6.2%; group D, −5%). No significant between-group differences were observed in total distance, training duration or ∑ HR zone training points. During the 8-week period the mean total running distances for groups N and D were 204±47 and 204±46 km respectively. Training duration was 1282±253 min and 1282±194 min and exercise scores based on ∑ HR zone training points were 4048±510 and 4073±624 respectively.

### Laboratory Measurements

IL-6 levels were below a detectable plasma concentration of 2 ng/L at all time points. No significant effect of time or group was found for CRP levels. Serum hepcidin changed over time and decreased in the recovery phase compared with baseline (p<0.05; Table 2).

**Table 2:** Mean values (±SD) of selected laboratory parameters measured at baseline, after eight weeks endurance training and after the recovery process in relation to baseline iron status.

							*Effect* p *value*	
*Measure*		*Group*	*Base*	*Train*	*Rec*	group	time	group* time
**Hepcidin (µg/L)**		**N**	190.09	203.37	92.61^a^	0.417	**0.004**	0.214
			(69.23)	(99.89)	(42.60)			
		**D**	173.53	134.87	102.89^a^			
			(83.07)	(84.79)	(27.40)			
**hsCRP (mg/L)**		**N**	1.04	0.77	6.80	0.580	0.906	0.276
			(0.57)	(0.35)	(14.86)			
		**D**	1,12	1,81	0,88			
			(1.28)	(3.32)	(1.41)			
RBC (*10^12/^L)		**N**	4.32	4.39	4.35	0.948	0.367	0.359
			(0.25)	(0.29)	(0.27)			
		**D**	4.41	4.38	4.24			
			(0.16)	(0.18)	(0.21)			
**Hb (g/L)**		**N**	132.43	134.71	132.71	0.742	0.390	0.314
			(6.75)	(5.50)	(3.04)			
		**D**	136.57	134.29	131.86			
			(6.60)	(6.60)	(7.93)			
Reticulocytes (*10^9^/L)		**N**	56.11	60.26	46.74	0.738	0.116	**0.021**
			(14.06)	(12.45)	(17.63)			
		**D**	48.36	61.17	60.74			
			(13.48)	(21.08)	(13.94)			
**TIBC (µmol/L)**		**N**	75.43	68.50^a^	69.71^a^	0.948	**0.016**	0.966
			(11.13)	(11.66)	(15.57)			
		**D**	74.71	68.40^a^	68.49^a^			
			(10.66)	(11.09)	(11.30)			
**TSAT (%)**		**N**	28.80	26.67	25.18	0.634	0.550	0.887
			(12.82)	(15.59)	(10.59)			
		**D**	32.59	25.02	28.57			
			(16.66)	(11.19)	(13.70)			
**Serum iron (µmol/L)**		**N**	21.71	17.26	18.37	0.663	0.262	0.903
			(10.23)	(7.50)	(9.96)			
		**D**	23.34	17.63	18.60			
			(8.94)	(10.78)	(6.32)			
**Ferritin (µg/L)**		**N**	33.00	22.14	31.71	0.006	0.067	0.700
			(14.07)	(8.03)	(27.84)			
		**D**	15.29	14.57	11.71			
			(3.68)	(10.68)	(2.69)			
**sTfR (mg/L)**		**N**	1.15	1.24^a^	1.22^a^	0.242	**0.008**	0.228
			(0.19)	(0.22)	(0.26)			
		**D**	1.27	1.33^a^	1.40^a^			
			(0.17)	(0.22)	(0.16)			
**sTfR-F index**		**N**	0.79	0.96^a^	0.95^a^	**0.03**	**0.014**	0.677
			(0.18)	(0.22)	(0.39)			
		**D**	1.1	1.32^a^	1.33^a^			
			(0.22)	(0.41)	(0.21)			
**Transferrin (g/L)**		**N**	3.23	2.86^a^	3.11	0.807	**0.000**	0.689
			(0.59)	(0.41)	(0.68)			
		**D**	3.2	2.73^a^	3.06			
			(0.59)	(0.49)	(0.43)			
**RDW (%)**		**N**	13.14	13.14	12.97	0.275	0.575	0.625
			(0.54)	(0.36)	(0.37)			
		**D**	13.91	13.46	13.63			
			(1.87)	(1.02)	(1.58)			
**Mean RBC Hb (pg)**		**N**	30.60	30.27^a^	30.10^a^	0.384	**0.004**	0.615
			(1.24)	(1.26)	(1.26)			
		**D**	31.20	31.04^a^	30.84^a^			
			(1.49)	(1.66)	(1.46)			
**Reticulocytes CHr (pg)**		**N**	33.03	31.16^a^	31.27^a^	0.828	**0.000**	0.798
			(1.32)	(1.06)	(1.13)			
		**D**	33.87	31.71^a^	31.94^a^			
			(1.71)	(1.9)	(1.49)			
**Hypochromic red cells (%)**		**N**	0,16	0.29^a^	0.50^a^	0.236	**0.001**	0.263
			(0.15)	(0.15)	(0.39)			
		**D**	0.11	0.30^a^	0.29^a^			
			(0.07)	(0.26)	(0.28)			
Leukocytes (*10^9/^L)		**N**	6.23	7.09	6.81	0.175	0.602	0.261
			(1.68)	(1.14)	(1.21)			
		**D**	5.91	5.67	5.93			
			(1.13)	(1.18)	(1.41)			
Platelets (*10^9/^L)		**N**	285.57	277.14	267.71	0.824	0.186	0.617
			(39.40)	(45.89)	(40.19)			
		**D**	289.43	264.86	275.14			
			(62.45)	(62.45)	(73.99)			

a significantly different from baseline value (p<0.05).

Base, Baseline; Train, Training; Rec, Recovery;hs-CRP, high sensitivity C-reactive protein; RBC, red blood cells; TIBC, total iron binding capacity; TSAT, transferrin saturation; RDW, red cell distribution width; MCH, mean cell Hb content of red cells; Reticulocytes CHr, Hb content of reticulocytes; sTfR, serum transferrin receptor; sTfR-F index, serum transferrin receptor: log serum ferritin; group N = baseline serum ferritin (SF) >20 µg/L, group D =  baseline SF ≤ 20 µg/L.

Prevalence of depleted iron stores defined by ferritin ≤20 µg/L increased to 71% (10/14) of the participants by the end of their training phase and remained higher than at baseline after the short recovery phase: 64% (9/14). However, when applying broader limits for ferritin levels in females according to classification by Custer et al [22] (see Methods), higher prevalence of depleted iron stores was observed: only two runners had ferritin levels >30 µg/L at baseline whereas 12 (86%) would have been catagorised as iron depleted. At the end of the training phase two participants developed ID, and after ten days of recovery iron stores were still diminished. One individual would have been considered as having IDA. Using the sTfR-F index resulted in the same individuals being classified as having ID at training. The classification of Custer et al and the sTfR-F index identified the same individual with IDA.

**Figure 1:**
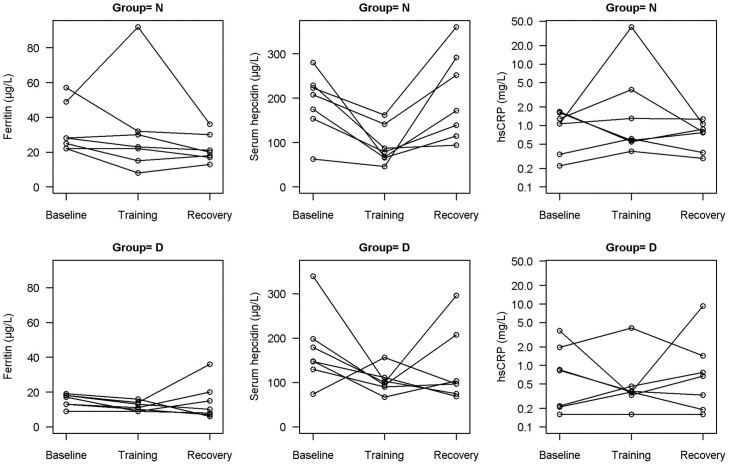
Individual values of ferritin, hepcidin and C-reactive protein within the study population, for all assessment points. Sorting according to ferritin level: group N = baseline serum ferritin (SF)>20 µg/L, group D = baseline SF<20 µg/L.

Closer observation of serum ferritin data would suggest excluding one individual in each group, who probably experienced an inflammatory response or illness seen as high hsCRP, which could influence the observed high ferritin levels (Figure 1). If both outliers are excluded, serum ferritin would be affected at training and recovery compared with baseline in both groups (time, group; p<0.05, both). Corrected values of ferritin at recovery in group N would be 21.67±9.04 µg/L and training in group D would be 11.54±5.44 µg/L. The group with initial low serum ferritin levels showed a smaller decrease (26% vs 33%) at the end of training (p = 0.053).

Temporal changes of laboratory parameters are presented in Table 2. Mean values for selected laboratory parameters, except ferritin in group D, were within normal levels throughout the study. Total iron binding capacity and the mean Hb content of red cells and reticulocytes decreased, whereas sTfR and the percentage of hypochromic red cells increased in training and recovery compared with baseline (time, p<0.05). Transferrin decreased only at training compared with baseline (time, p<0.05). There was a group*time interaction (p<0.05) for reticulocytes. Group N experienced a decrease in reticulocytes at training, since in D group reticulocytes increased compared with baseline (p<0.05, both cases). The correlation between hepcidin level, iron status indicators and biomarkers of inflammation measured at baseline, after long-term training and after ten days of recovery can be found in Table 3. Also, change in haemoglobin or ferritin level and incidence of anaemia do not correlate to group allocation or index activity score (p>0.3 for all).

**Table 3:** Correlation between hepcidin level, iron status indicators and biomarkers of inflammation measured at baseline, after long-term training and after ten days of recovery.

	Hep (µg/L)			Hep (µg/L)			Hep (µg/L)		
Time	Base			Train			Rec		
Group	N	D	Whole	N	D	Whole	N	D	Whole
**Hemoglobin (g/L)**	−0.01	0.35	−0.18	0.47	−0.51	−0.06	−0.20	−0.40	−0.26
**Ferritin (µg/L)**	−0.84*	−0.09	−0.26	0.22	−0.70	−0.09	0.04	0.07	−0.11
**TSAT(%)**	−0.08	−0.40	−0.26	−0.17	−0.28	−0.19	0.24	0.26	0.27
**sTf-R (mg/L)**	0.46	−0.24	0.11	0.40	−0.29	0.22	0.20	0.47	0.38
**RDW (%)**	0.40	−0.09	−0.00	−0.15	0.56	0.22	−0.33	0.24	0.11
**CRP (mg/L)**	0.48	0.16	−0.3	−0.82*	−0.72	−0.60*	−0.17	−0.22	−0.26

Hep; hepcidin; Base, Baseline; Train, Training; Rec, Recovery; TSAT, transferrin saturation; sTfR, serum transferrin receptor; RDW, red cell distribution width; hs-CRP, high sensitivity C-reactive protein.

Pearson correlation;

* p<0.05.

## Discussion

Our study shows that 50% of female runners who entered the study already had depleted iron stores, which increased to 71% at the end of eight weeks’ endurance running. Concentrations of hepcidin tended to be lower during long-term running training and were reduced after a recovery phase, regardless of iron status at the start of the study. We also observed increased values of the sTfR-F index and sTfR after training and recovery phase.

Iron deficiency is indeed a relevant issue to influence exercise performance among female athletes and is potentiated through physical training. Without a widely accepted criterion at hand, the prevalence ranges from 40% to more than 80% [2], [25], [26]. However, serum ferritin levels below which athletes are considered “iron deficient” vary in different studies [2], [21], [26]. We found that our training protocol increased the risk of depleting iron stores: 42% of runners in the group with depleted iron stores at baseline developed ID or IDA regardless of the criterion used. In all of them, the ferritin values decreased below 8 µg/L. Although it is reported that the sTfR-F index is a more reliable marker of ID and remains stable in athletes despite significant day-to-day changes in either ferritin or sTfR, it appears that the sTfR-F index did not identify early functional iron depletion, as previously reported in athletes whose serum ferritin levels were marginal but did not fall below the threshold for ID [3], [23], [27]. Recent data show strong associations between serum hepcidin and both serum ferritin and the sTfR-F index [28], [29].Similar association was as observed in our study, when we excluded two individuals with elevated CRP. Importantly, a high degree of intraindividual response in hepcidin concentration requires careful interpretation of findings [28].

Another clinically relevant finding is that a short recovery phase was insufficient to normalize ferritin values as recently reported [4]. Since 64% of the runners remained at low levels of iron stores, our exercise protocol induced undesirable effect. It has to be noted that general recommendation of physical exercise as healthy behaviour to reduce several diseases may also have potential for unwanted effects. The changes of some other iron-related parameters in our study, such as sTfR, mean red cell Hb, hypochromic red cells, reticulocytes and mean reticulocyte Hb, showed increased erythropoietic activity but reflect insufficient iron for normal erythropoiesis; this was observed independently of ferritin level. It is therefore likely that a prolonged training phase would probably further deplete iron stores and could lead to IDA with a negative impact on athletes’ performance [6], [30].

We found that hepcidin concentration decreased during long-term running training, regardless of iron status at the start of the study, which is not as previously reported by Karl et al [17]. Importantly, they reported lower serum hepcidin concentrations in female soldiers with IDA, which is in line with significant changes of hepcidin in our participants, as almost two third of them had low ferritin level in recovery phase. However, we observed increased sTfR concentrations after training as in study previously mentioned [17]. It appears that the decreased levels of hepcidin in our study could be more associated through its homeostatic regulation due to iron demand rather than due to exercise-associated inflammation as no evident inflammation was detected. A recent finding suggests that there may be a difference in activity of acute post-exercise urine hepcidin response when iron stores are compromised [15]. In our study there were no differences in the serum hepcidin dynamic in female runners with depleted and normal iron stores. In the study mentioned previously [15] participants had ferritin levels <35 µg/L, as in all our participants at training, which could possibly explain the non-significant difference between our groups. On the other hand we may not compare the results in a straightforward manner, since it is known that serum hepcidin does not correlate with urinary hepcidin [14], [31]. As reported in previous research [14]–[16], it appears that there is a varied individual hepcidin response (Figure 1). Additional caution is needed when comparing the results across studies which have used different assays for hepcidin detection because reference ranges or units may vary considerably.

Although we did not observe changes in markers of inflammation, our results and those recently reported by Karl et al [17] do not preclude the possibility that repeated bouts of high intensity exercise may provide an acute stimulus resulting in transient increases of IL-6 followed by elevated hepcidin and may influence iron disruption as observed in earlier studies [12], [13], [15].

We are aware of potential limitations. Firstly, a sample of 14 participants may not be sufficient to show potentially relevant changes in markers of iron status dynamics over time, partially due to variabilty assocaited statistical issues rather than true physiological effects. This limitation is design driven as field studies usually recruit up to 20 participants [12]–[16], with only rare exceptions [17]. It also remains to be determined whether our time points were completely appropriate for evaluation of short- and long-term responses. Both these issues, however, are shared with other reports and there is an absence of straightforward guidance for clinical practice. Focusing on female athletes is physiologically driven and based on a higher prevalence of ID among them. Nonetheless, in view of emerging reports, focusing on such a group can be misleading and may be considered as potential limitation [4].

In conclusion, on the basis of our results and those of previous studies we can confirm the relation between decreased hepcidin and iron stores in female athletes after long-term endurance running [17], [29]. Further studies are required to elucidate the cumulative effects of repeated high-intensity exercise bouts on inflammation, hepcidin and iron status in an active population.

Our findings, together with previous reports, stress the importance of ID in female athletes. This is a multifaceted issue and awareness among athletes, trainers and medical staff is of utmost importance. Simple parameters such as serum ferritin and transferrin saturation appear sufficient to identify most of the ID in the majority of athletes, but, whether reference levels commonly accepted for general population are applicable in this study population remains controversial. In athletes, tissue iron deficiency without anemia may have a negative impact on both oxygen transport and utilization [32].Thus, for top-level athletes it may be more reasonable to apply criteria used in diseased populations in order to prevent any iron deficiency related physical exercise limitations [33]. This is clinically important because both in patients [33], [34] and in athletes [35], [36], iron supplementation improves their performance. In any case, further research in terms of sampling and established or emerging biomarkers is warranted.
